# Co-Management of COVID-19 and Heart Failure During the COVID-19 Pandemic: Lessons Learned

**DOI:** 10.31083/j.rcm2306218

**Published:** 2022-06-16

**Authors:** Alberto Palazzuoli, Carl J Lavie, Paolo Severino, Amardeep Dastidar, Eva Sammut, Peter A. McCullough

**Affiliations:** ^1^Cardiovascular Diseases Unit Department of Medical Sciences, Le Scotte Hospital University of Siena, 53100 Siena, Italy; ^2^John Ochsner Heart and Vascular Institute, Ochsner Clinical School, The University of Queensland School of Medicine, New Orleans, LA 70121, USA; ^3^Department of Clinical, Internal, Anesthesiology and Cardiovascular Sciences, Sapienza University of Rome, 00161 Rome, Italy; ^4^University Hospital Bristol and Weston NHS Foundation Trust, BS1 3NU, UK; ^5^North Bristol NHS Trust, BS1 3NU, UK; ^6^Truth for Health Foundation, Tucson, AZ 85728, USA

**Keywords:** heart failure, SARS-CoV-2, outbreak, diagnosis, management

## Abstract

The COVID pandemic has brought many new challenges worldwide, which has impacted on patients with chronic conditions. There is an increasing evidence base suggesting an interaction between chronic heart failure (HF) and COVID-19, and in turn the prognostic impact of co-existence of the two conditions. Patients with existing HF appear more prone to develop severe complications on contracting COVID-19, but the exact prevalence in patients with mild symptoms of COVID-19 not requiring hospital admission is poorly investigated. In addition, hospitalization rates for acute HF over the pandemic period appear reduced compared to previous periods. Several key issues remain rather unaddressed and, importantly, a specific algorithm focused on diagnostic differentiation between HF and acute respiratory distress syndrome, a severe complication of COVID-19, is still lacking. Furthermore, recent data suggests potential interaction existing between HF treatment and some anti-viral anti-inflammatory drugs prescribed during the infection, raising some doubts about a universal treatment strategy for all patients with COVID-19. With this manuscript, we aim to review the current literature in this field in light of growing understanding of COVID-19 in the setting of the HF population, its associated morbidity and mortality burden, and the impact on healthcare systems. We hope that this may stimulate a discussion to guarantee a better, more tailored delivery of care for patients with HF in the setting of concomitant COVID-19 infection.

**Highlights**

- Few studies specifically evaluated the exact impact of HF and prognostic implications in infected patients; Similarly the exact prevalence and consequences of COVID in HF patients, remains unexplored.

- The modality of HF occurrence and pathophysiological mechanisms causing acute HF during infection encompass micro and macro vascular coronary damage, direct myocyte injury, systemic inflammation and endothelial dysfunction, but it is not know the exact HF ethiology leading to HFrEF or HFpEF.

- A precise diagnostic algorithm capable to differentiate patients presenting with acute dypnea before to have swab response is lacking and should be based on simple clinical laboratory and chest diagnosticprocesses.

- Carefull attention should be deserved in patients with both HF and COVID infection to avoid potential arrythmic and heamdynamic consequences.

## 1. Introduction 

Severe acute respiratory syndrome coronavirus (SARS-CoV)-2, the cause of the COVID-19 illness, firstly observed in Wuhan, China, in December 2019 and spread to other areas worldwide, rapidly reaching pandemic levels. The COVID pandemic has brought many new challenges with substantial healthcare disruption including unprecedented curtailment of elective work, transition to remote consultations and significant fluctuations in referral patterns to hospitals. The high rate of symptomatic transmission has presented unique dilemmas in attempts to contain the disease driving national lockdowns and prompting shielding of vulnerable groups with significant economical and societal impact. Failure of the COVID-19 vaccines to provide substantial protection against the Delta and other mutant strains has made it clear that COVID-19 will continue to be a coexistant clinical problem with heart failure (HF) that requires early treatment in order to reduce the risks of hospitalization and death. The disruption to healthcare has inevitably and particularly affected patients with many chronic health conditions [1, 2].

Whilst most people contracting COVID-19 appear to have an initally mild burden of symptoms of cough and fever, worsening to a modest infection with symptoms mirroring influenza, the acute phase of COVID can cause pneumonia and respiratory failure with associated significant mortality [3]. Infection with COVID-19 also appears to bring additional specific features in some, which include increased thromboembolic risk, leading to deep vein thrombosis, stroke and myocardial infarction. Given that studies have focused on the more severe end of the disease spectrum and that the majority of infected patients do not require admission to hospital, the true prevalence remains unknown. Thus, patients with HF should receive where available intravenous administration of monoclonal antibodies (e.g., casirivimab and imdevimab) against the SARS-CoV-2 spike protein followed by sequenced multidrug therapy with a protocol to best manage viral replication, cytokine storm, and possibly to prevent immune thrombosis events [4].

A recent large database study showed an higher risk for hospitalized HF patients and COVID-19 diagnosis, with nearly 1 in 4 dying during hospitalization [5]. In parallel, an Italian registry also studied mortality in hospitalized HF patients contracting COVID-19 and found this was associated with significant risk for multi organ complications [6]. Although initial studies showed that patients specifically affected by chronic HF are classified as at high risk, HF has often been included among all the other cardiovascular disease (CVD) causes of admission, and few early studies specifically evaluated the exact impact of HF and prognostic implications in infected patients [7]. Given the high rates of symptomatic infection, the exact prevalence of HF patients, and indeed of total cases of COVID-19 remains incompletely unexplored. Additionally, thorough investigation of whether HF patients contracting COVID-19 develop a more severe clinical manifestations because of direct cardiac damage or because of their frailty related to systemic and metabolic associated diseases, remains incomplete. In order to address the relationship existing between COVID-19 and HF, and to provide specific recommendations, the European Society of Cardiology and Chinese Heart failure societies have recently developed a joint document focused on the management for patients with both diseases [8]. Finally, exploration of interactions between HF and COVID-19 is hampered in subjects presenting with acute dyspnoea as it can be challenging to distinguish between those with SARS related COVID manifestations and those with acute HF related symptoms. A standardized diagnostic screening approach focused on diagnostic differentiation between acute HF (AHF) and SARS related COVID-19 infection is lacking. Finally, some discordances exist in patients with both HF and virus infection: some reports showed a worse outcome in patients with both HF with reduced ejection fraction ( HFrEF) and preserved ejection fraction (HFpEF) [9, 10], whereas a recent paper suggests low EF is related with adverse event increase [11]. In this current manuscript, we aim to review literature focusing on the interplay of HF and COVID to prompt interventions that might guarantee a better management and outcomes for patients with HF and COVID-19.

## 2. Direct Cardiac Effects of COVID-19 Infection

It has become clear that cardiac involvement is not uncommon in the setting of COVID-19 infection with biomarkers of cardiac injury and stress, such as natriuretic peptides and troponin, found to be elevated in about 25% of those with a severe COVID-19 infection and associated with a bad prognosis. Several studes have demonstrated acute cardiac injury such as myocarditis, myocardial stress and cardiomyopathy [12, 13, 14, 15]. Furthermore, early work demonstrated that patients with pre-existing cardiac conditions are more susceptible to severe COVID manifestations however, it is unclear if their elevation reflects older age, the high prevalence of CVD risk factors and pre-existing CVD of those requiring hospitalisation, or direct effects of the viral insult [16, 17]. Some have suggested that COVID-19 might invade the myocardium and cause direct damage to myocytes. Possible proposed mechanisms for this include a key role of angiotensin receptors in the inflammatory response [18]. Like the SARS-COV virus, the SARS-COV-2 virus gains cell entry via binding of its transmembrane spike protein to host endogenous angiotensin converting enzyme (ACE)-2 proteins. ACE2 is a homolog of ACE which, like ACE, acts directly on the renin-angiotensin-aldosterone system. Binding of the virus to the ACE2 receptor induces downregulation of ACE expression which may result in unopposed angiotensin II accumulation and local renin-angiotensin-aldosterone system (RAAS) activation. This has the potential to exacerbate tissue injury and promote inflammation and thrombosis. In the setting of HF, where there is known maladaptive activation of the RAAS with increased circulating levels of ACE2 secondary to reduced cardiac output, there has been speculation that the biochemical environment commonly seen in the setting of HF may increase susceptibility to COVID and could lead to a more severe clinical course of the infection.

**Fig. 1.**
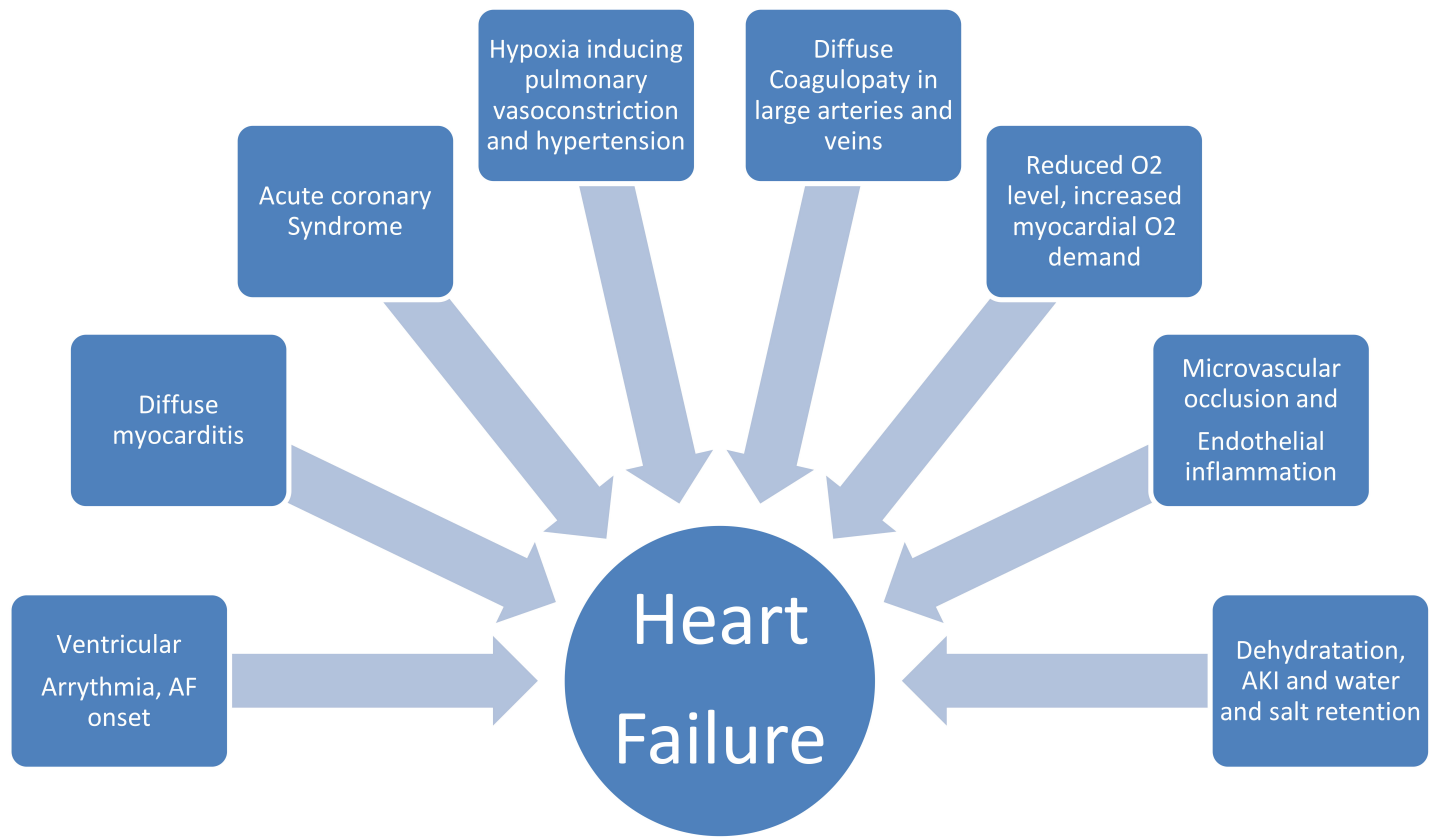
**Potential Mechanisms leading to Cardiovascular complication induced by infection, systemic inflammatory and endothelial dysfunction may impair stable HF condition or promote a new HF onset**.

There are a few additional reasons to explain the association between cardiac manifestations and, specifically, HF and poorer outcomes in COVID-19. Any severe infection might cause tachycardia, increase myocardial oxygen demand, and worsen cardiac function. Coexisting hypoxemia due to ARDS might impair oxygen transport and delivery at the myocardium and peripheral muscles, potentially triggering ischaemia, acidosis and oxidative stress. COVID-19 can also cause coronary spasm or plaque rupture, or endothelial inflammation with microvascular obstruction and additional myocardial damage. Endothelial dysfunction and increased thrombogenic activity, which are common in patients with HF, might be aggravated by a COVID-19 infection and subsequent immobilization, and cause ischaemia or infarction in the brain or other organs. Dehydration might cause hypotension and renal dysfunction, which may further enhance the activity of the RAAS. Development of pulmonary hypertension secondary to SARS might aggravate symptoms, causing right ventricular (RV) strain and dysfunction. Finally increased thrombogenic activity may increase the risk of pulmonary embolism leading to sudden right-sided HF [19, 20]. Additionally, simultaneous acidosis or electrolyte unbalance and myocardial inflammation may become potential triggers for malignant arrhythmias [21]. The reduced arrhythmogenic threshold may be also exacerbated by the use of some anti-inflammatory drugs with potential interference with electromechanical activity such as QT prolongation (i.e., hydroxychloroquine, clarithromycin; Fig. 1).

### 2.1 Impact of HF on COVID Severity

A number of studies have explored the impact of pre-existing HF, or the presence of ventricular dysfunction, in terms of outcome from COVID infection. In France, Matsushita *et al*. [22] performed a retrospective single centre telephone study involving patients who had undergone percutaneous coronary intervention (PCI) for acute coronary syndrome (ACS) between 2014–2018. They divided the population into those with a left ventricular (LV) ejection fraction (LVEF) <40% (91 patients), or >40% (798 patients) at the time of PCI. The incidence of COVID-related hospitalization or death was higher in patients with pre-existing LVEF <40%. However two US studies did not confirm a poorer outcome in HF patients regardless EF [10, 11]. In an Italian multicentre registry study, Tomasoni *et al*. [6] reported data from a multicentre registry study on 692 patients who had tested positive for COVID. Mortality was higher in those with known HF compared to no HF even after adjustment for variables. Higher in hospital complications, including acute HF, acute kidney injury, multi-organ failure and sepsis.

Rumery *et al*. [23] in the USA studied a large group of, predominantly male, veterans with and without pre-existing HF. They noted a higher 30 day mortality and admission rate with COVID in those with known impaired LVEF <45%. Lassen *et al*. [24] prospectively evaluated echocardiographic parameters in patients with COVID-19 compared to controls. They found significantly reduced global longitudinal strain (GLS), LV diastolic function, and RV function in COVID cases. Those with LV/ RV dysfunction were more likely to die from COVID. Tricuspid anular peak systolic excursion (TAPSE), LVEF and GLS on univariate analysis was associated with an increased risk of death and TAPSE and GLS remained significant on multivariate analysis even with exclusion of those with pre-existing HF.

### 2.2 Impact of COVID and Pre-Existing HF on HF Admissions 

With the impact worldwide on the provision of healthcare and restrictions, there have been clear temporal changes in referral patterns to hospitals over the course of the pandemic. Numerous studies, spanning the globe, have demonstrated a reduction in admissions to hospital with AHF, in line with other emergency presentations, such as ACS during the peaks of the pandemic [25, 26, 27, 28, 29, 30], Collectively, these studies demonstrated a reduction in AHF admissions at the time of peak of the pandemic compared to corresponding periods the previous year or prior to the pandemic which later returned to normal/near-normal as COVID cases dropped. This suggests a reluctance at referrer or patient level to attend hospital until deemed absolutely necessary. Indeed, one study demonstrated a relative increase in admissions to hospital following media reports encouraging patients to seek medical attention if necessary [30]. Furthermore, this is supported by data showing that the severity of AHF in terms of LVEF and NYHA class was more advanced in those admitted [24]. The longer-term consequences of this reduction in attendance to hospital remains to be seen, however, in an interesting small study in Austria by Sulzgruber *et al*. [31] they demonstrated a reduction in ACS admissions in the period immediately after the outbreak compared to before and in parallel, with a delay of around two weeks, they reported a rise in admissions with AHF suggesting that delayed presentation to hospital with ACS led to symptomatic ventricular impairment. Some have demonstrated an increase in out of hospital arrests in the pandemic period which may relate to these delays in presentation to hospital [32, 33].

In addition, the rapid surge in COVID-19 admissions critically impacted on healthcare delivery and did not spare hospital staff, causing widespread shortage of doctors and nurses, and the need to redeploy members of the cardiology team to help with acute emergencies. With increasing COVID infections, patients admitted with HF were less likely to be admitted in cardiology wardsand were more likely to have their treatment withdrawn [25, 34, 35]. The effect of this again has not been fully evaluated to date however, the lack of specialist input may have impacted on outcomes and suggests that patients with HF were unable to receive appropriate medical attention until their clinical condition was extremely compromised. Importantly, due to an older age, a greater number of comorbidities, and the likelihood or a poor survival, many patients with heart failure may have been denied admission to intensive care unit or were not considered for invasive treatments such as mechanical ventilation, which might have also contributed to the increased mortality [36, 37].

Studies in the setting of AHF have also demonstrated worsened outcome since the onset of the pandemic which is likely to be multifactorial and related to the points discussed above. Doolub *et al*. [28] examined short-term (30-day) mortality and reported despite similar demographics in the pre- versus post COVID groups, that age and COVID status were independent predictors of mortality, driven by positive COVID status (Table 1, Ref. [9, 25, 26, 27, 28, 29, 30, 32, 33, 34, 35, 36, 37]).

**Table 1.** **Epidemiological studies investigating HF hospitalization during the lock down period: despite a reduction in HF related hospitalization, admitted patients experienced a more severe disease and complications**.

First author	Observational period	Cohort	Methods	Main findings
Andersson C. *et al*. [30]	January 1 to March 11	Danish national data	Incidence of HF hospitalization before and after the lockdown and mortality for HF comparing Jan-March 2019 with March 2020	new-onset HF diagnoses and HF hospitalizations for worsening HF were significantly lower in 2020 vs 2019 (0.63 versus 0.99 per 1000 person-years). Mortality was similar before and after the national lockdown
Cannata C. *et al*. [26]	January 7 to June 14	South London hospitals UK	Observational study comparing data from 7 January to 14 June 2020 with those of the same period in 2019	Significant reduction in hospitalizations during the COVID-19 peak, followed by a return to 2019 levels. Increased in hospital mortality compared to previous period
Frankfurter C. *et al*. [34]	March 1 to April 19	Toronto Hospital CN	Hospitalization events for acute HF from March 1, to April 19, 2020 and 2019 in an urban hospital	Decrease in ADHF-related visits and admissions was observed (8.6%–78.5%, *p* = 0.009). A trend toward an increase of in hospital mortality during infection surge compared with 2019
Bromage DI. *et al*. [25]	March 2 to April 19	King’s College Hospital, London	National Heart Failure Audit for England and Wales, between 2 March–19 April 2020 were compared to same period in 2017 to 2019	A significantly lower admission rate for AHF was observed during the study period compared to all other periods (4 vs 10.5 weekly admission in COVID vs pre COVID period), but hospitalized patients had more severe symptoms at admission
Cox Z. *et al*. [29]	March 22 to April 20	Vanderbilt University Medical Center, US	AHF hospital census from March 22nd to April 20th 2020 relative to the same calendar day in 2019	Decreased number of hospitalization compared with same period of previous year (–11 ± 12% vs –46 ± 16%)
Rey JR. *et al*. [35]	March 1 to April 20	Madrid Hospital Spain	Mortality rate in patients with COVID-19 infection and a prior diagnosis of HF between 1 March and 20 April 2020	Infected COVID-19 patients with history of CHF are prone to develop acute decompensation. Patients with CHF showed higher mortality rates (48.7% vs 19.0%)
Bhatt AS. *et al*. [9]	January 1 to March 30	Retrospective analysis from Mass General Brigham system US	Premier Healthcare Database to identify patients with at least 1 HF hospitalization or 2 HF outpatient visits between January 1, 2019, and March 31, 2020	A total of 23,843 patients were hospitalized with acute HF, 6.4% were hospitalized with COVID-19. 24.2% of patients hospitalized with COVID-19 died in-hospital compared to 2.6% of those without infection
Chatrath N *et al*. [36]	March and 6 May 2020	Retrospective single center study examining patients with chronic HF admitted in London hospital	In-hospital mortality assessment in patients with chronic HF and associated COVID-19 infection	Patients with HF and associated COVID-19 had a significantly increased inpatient mortality compared with hospitalized HF patients without infection (50% vs 10.6%)
Baldi E *et al*. [32]	February 21 to april 20	Lombardia region Italy	Lombardia Cardiac Arrest Registry measuring out-of-hospital cardiac arrests from February 21 through March 31, 2020 with those that occurred during the same period in 2019	362 cases of out-of-hospital cardiac arrest were identified, as compared with 229 cases identified during the same period in 2019 Out hospital Cardiac arrest occurred much more during pandemic period with 52% increase compared with 2019
Marijon E *et al*. [33]	March 16 to April 26	Observational registry from Paris, France	observational study using data for extra hospital cardiac arrest, systematically collected since May 2011	the maximum cardiac arrest incidence weekly increased from 13.42 to 26.64 per million inhabitants, in the final weeks of the pandemic period; the proportion of patients who had cardiac arrest admitted alive decreased from 22.8% to 12.8% in the pandemic period
Doolub G *et al*. [28]	7 January to 27 April	South west England UK	single-centre observational study, examining referrals to the acute heart failure team over a period between 7 January to 27 April 2020	Early period reveals a reduction in hospitalization and mortality respect to late period. The 30 day case fatality rate was increased by 10% during late period
Severino P *et al*. [27]	21 February to 31 March	Multicenter retrospective Italian study	retrospective analysis on HF admissions at eight italian hospitals throughout 21 February to 31 March 2020, compared with an inter-year period (21 February to 31 March 2019) and an intra-year period (1 January to 20 February 2020)	Significant hospitalization reduction compared with previous year. Admitted patients were in more advanced NYHA class; mean admission rate during the case period was 2.80 per day, compared with intra-year period 3.94 per day; or with inter-year 4.92 per day
Christensen DM *et al*. [39]	December 17, 2020 to January 2021	nationwide Danish study	Nationwide survey identifying all first-time admissions for HF, Ischemic heart disease Ischemic stroke and atrial fibrillation during first five weeks of the second Danish lockdown	Incidence of new-onset heart failure and atrial fibrillation remained stable compared tom the previous year, with significant drop in new-onset ischemic heart disease and stroke

Finally, a number of studies have demonstrated impact of pre-existing HF and development of AHF in relation to outcomes during the COVID pandemic. In the study, by Rey *et al*. [35] in Spain over 3000 patients with confirmed COVID-19 infection were analysed. Patients with a previous history of HF were more prone to the development of AHF (11.2% vs 2.1%; *p *< 0.001) and had higher levels of N-terminal pro brain natriuretic peptide. Patients with previous HF had higher mortality rates at 30 days. Arrhythmias during hospital admission and HF were the main predictors of AHF and patients developing AHF had significantly higher mortality. Chatrath *et al*. [36] investigated the impact of concomitant COVID 19 infection in patients in hospital with pre-existing HF. COVID-19 infection resulted in significantly increased mortality in hospital with more chance of acute kidney injury or myocardial injury. In Brazil, Bocchi *et al*. [38] performed a small retrospective study on 16 patients with advanced systolic HF comparing a group admitted with HF who then developed COVID in hospital versus those admitted with HF admitted with COVID. They noted a worsening of HF with COVID with more ionotropes/need for intra-aortic balloon pump or intensive care and overall a high mortality rate. Interestingly, they noted presentation of worsening HF with COVID infection was of haemodynamic compromise rather than fever or signs of systemic infection.

### 2.3 Impact of COVID on the Stable Chronic Population 

The pandemic has brought worldwide disruption to the provision of healthcare services for patients with chronic conditions, such as HF. There has been curtailment of elective hospital investigations and treatment, including outpatient clinic review and home visits, with changes to pathways in the acute setting. Access to primary services was also limited to the most severe and urgent cases, with a massive reduction in referrals for CVD consultations [39]. The nature of the pandemic has driven a transition to remote consultations to facilitate perceived safer review of non-emergency care. Given the burden on these services by patients with a diagnosis of HF, this has been studied specifically in this context by a number of groups. In France, Chague *et al*. [40] investigated the impact of national lockdown in patients with known congestive HF. They investigated patients in the outpatient setting by telephone during the lockdown in France. They noted increased psychological stress and worsened symptoms, reduced physical activity particularly in women and those living in urban areas. Other lifestyle patterns were also altered with weight gain common, tobacco use in smokers increased, and a reduced adherence to salt and water restrictions. The study noted good adherence to restrictions and reported no disruption to access to medications. In the UK, a similar questionnaire-based study with 1050 respondents reported higher anxiety levels regarding COVID than HF, a reluctance to attend hospital and some disruption to appointments and medication provision services [41]. Many hospitals cancelled cardiology out-patient clinics, home visits and elective operations, and postponed important diagnostic investigations. Access to primary services was also limited to the most severe and urgent cases, with a massive reduction in referrals for CVD consultations [39]. All of this might have led to sub-optimal management and under-diagnosis of new HF cases in the community and to a decreased rate of HF hospitalizations, and may have contributed to a substantial increase in the rate on fatal adverse CVD events, in hospital and in the community.

The strategies put in place to limit spread of the dinfection, have resulted in patients with chronic conditions not receiving face to face specialist input for far longer than was expected at the outset. Many did not have their treatment optimised, and as a result may have deteriorated. As the burden of the pandemic starts to ease in general terms, there is now a need to understand that COVID-19 may have a permanent presence and a balance must be stuck to enable patients to obtain necessary reviews while avoiding unnecessary infections. In contrast however, already stretched healthcare systems have developed novel strategies to deal with patients remotely, such as video consultations and home telelemonitoring which, coupled with in-person care, may result in more regular contact and ultimately benefit patients living with chronic conditions. Recent experience in New York with a multisensor device may suggest the beneficial effects of current approach in reducing both hospitalization and infection [42]. Furthermore, many patients, faced with restrictions have taken more ownership of their health and well-being and this can only be a good thing in a wider sense (Table 1).

## 3. How Can We Differentiate AHF and Acute Respiratory Distress Syndrome (ARDS) in COVID Patients? 

Distinguishing between these two conditions may be difficult in absence of specific diagnostic criteria; a rapid and customized algorithm may help clinicians at the bedside deliver optimal care. A detailed history and clinical examination can be very useful for diagnostic differentiation. Up to 80% of patients admitted with COVID-19 have, or have recently had, fever, which often resists to antipyretic treatment, and gastrointestinal symptoms. The recent onset of exertional breathless, persistent cough and orthopnea would suggest an infection, but does not exclude HF. Isolated pulmonary crackles or diffuse reduction in pulmonary ventilation associated with tachypnea, are much more suggestive for a respiratory infection, not necessarily secondary to COVID-19, and blood cultures should be obtained in those who are febrile. In those with prevalent HF, clinical examination usually reveals sign of pulmonary and systemic congestion; the identification of a murmur or a third heart sound on auscultation should suggest a cardiac aetiology. A carefully collected past medical history is paramount, as known cardiovascular risk factors or established disease would identify those more likely to develop HF and to have a poorer prognosis if infected by COVID-19 [43]. Unfortunately during the early phase it can be challenging to obtain a detailed history from patients who are acutely short of breath alongside the restraints of personal protective equipment and ward isolation [44]. Limitations such as patient distress, confusion, anxiety are additional hurdles.

A normal chest X-ray would not exclude a COVID-19 infection or HF; cardiomegaly or frank pulmonary oedema should not be missed and should prompt an echocardiogram. When in doubt, a CT chest might demonstrate bilateral interstitial pneumonia, which is a clear sign for COVID-19 infection, with or without pulmonary embolism, one of its frequent complications. Identification of pleural effusion might be common in both conditions, but not specific [45].

A simple electrocardiogram is an important source of information. Indeed, signs of a previous myocardial infarction or a prolonged QRS might suggest underlying cardiac dysfunction and greater risk of complications. Other findings, such as tachycardia or atrial arrhythmias, are common in both ARDS and HF, and might not be helpful in differentiating between the two conditions, although they have therapeutical implications [46]. A mild elevation of natriuretic peptides have been described in COVID infection and are thought related to the direct multi organ damage related to infection, cytokines overdrive, or increased pulmonary pressure and therefore are not always a marker of concomitant HF per se but significantly increased natriuretic peptide levels are felt to be more consistent with AHF than COVID-related SARS. Low levels of natriuretic peptides exclude HF and suggest a good outcome even amongst those diagnosed with COVID-19. Elevated levels of inflammatory markers (C-reactive protein or ferritin) associated with relative lymphopenia raise the clinical suspicion of a COVID-19 infection [47]. Troponin can be elevated in patients admitted with HF, as well as in those with COVID-19, and suggest a greater risk of CVD and non-CVDcomplications [16]. Hypoxaemia and hypocapnia associated with low oxygen saturation below 90% and respiratory acidosis are specific signs of respiratory distress or related thromboembolic complication; conversely, hypoxaemia without hypercapnia and relative acidosis or respiratory alkalosis are more typical for HF. D-dimer and fibrinogen, reflecting activation of both haemostatic and fibrinolytic systems, would be of aid to identify those with a higher risk of thrombo-embolic events and death, but not to differentiate between the two conditions [45, 47].

**Fig. 2.**
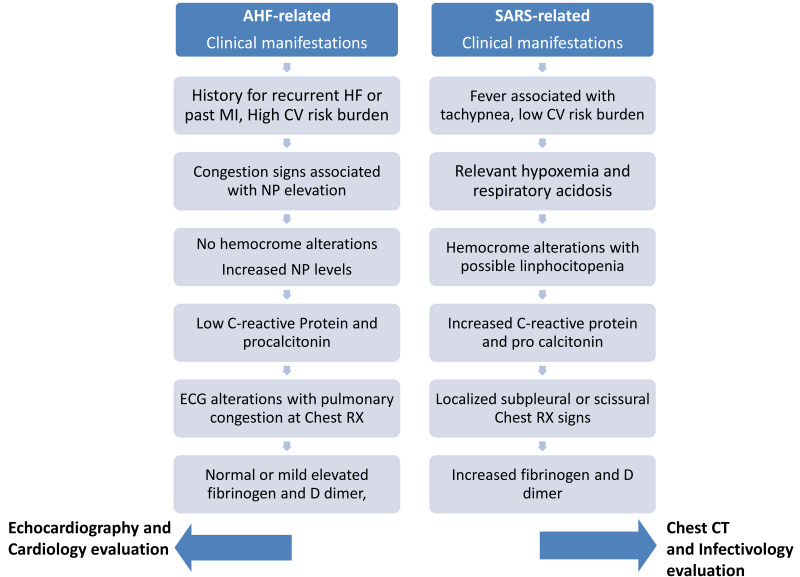
**A specific diagnostic algorithm addressed to the early recognition of acute dyspnea due to respiratory or cardiac manifestations**.

Echocardiography should always be performed when HF is suspected, as it might identify substantial LV systolic dysfunction or valve disease, helpful to guide ongoing management [48]. Signs of fluid and pressure overload (i.e.,: a dilated inferior vena cava or pulmonary hypertension) or RV dilatation and dysfunction, would indicate the need for additional investigations to evaluate both lung parenchyma and vessels [49] (Fig. 2).

Absence of cardiac dysfunction at imaging and of B-lines on lung ultrasound would exclude ARDS and HF, and suggest to look for alternative causes for breathlessness (i.e.,: anxiety, asthma, or exacerbation of chronic obstructive lung disease). After initial assessment on the basis of single patients score addressing for AHF or for ARDS patients may follow a different route and management. In order to accelerate diagnosis and start customized treatment patients with high scores for respiratory involvement may be carry out chest CT even before swab results, to evaluateevenctiual pulmonary injury, its extension location and fibrotoic evolution. Converesely, if diagnoasi portrend for HF a tailored therapy woith diuretics inotropes and vasodilators may be started, without further procedures. Current picture analyzed the distinct process related to diagnostic differentiation, however COVID infection may be the trigger for HF onset. Unfortunately, whether COVID infection is the primary trigger for LV diastolic or systolic dysfunction is currently ignored and detailed imaging evaluation may be encouraged in both hospitalized and non-hospitalized positive subjects.

Notably, an echocardiographic and magnetic resonance combined analysis, showed that an elevated percentage of infected subjects with elevated troponin, had cardiac involvement in terms of reduced ventricular strain and myocardial edema, even with minimal symptoms [50]. Nevertheless this screening cannot be extended to the whole population, and current findings are probably analogue to other viral infections, in which myocardial involvement has been less investigated. Recently, the use of hand held echocardiographyic devices has been proposed as an in-expensive, rapid, and quick screen for cardiac abnormalities in COVID-19. Therefore, the combination of cardiac and chest ultrasound evaluation may provide a better definition of underlying disease [51]. Alteration. Similarly, the contemporary rectruitment of echocardiographic abnormalities and elevated troponin suggests a very bad prognosis in COVID-19 [52].

## 4. In-Hospital Treatment Dilemma

Adverse clinical outcome seen in this setting may be due to the lack of specific management of patients with HF presenting with COVID-19. Drugs employed to manage cytokine storm, including inhaled budesonide, oral/intravenous corticosteroids, and colchicine all have supportive randomized trials irrespective of their patient treatment venue. The use of recombinant humanized anti-interleukin-6 receptor monoclonal antibody (in patients with rapid respiratory decompensation) have been shown to provide some benefit [53]. Several concerns arise from current antiviral and antiflammatory drugs commonly employed during severe infection and diffuse pneumonia. Indeed, the beneficial effect of remdesivir, tolicizumab and corticosteroids remain questioned due to contrasting results and to the restricted number of randomized clinical trial and different population tested [54, 55]. Moreover in patients with HF there are no specific studies demonstrating the effective benefit. and some of the treatments trialled for treatment of COVID-19 can exacerbate HF. Corticosteroids are known to increase fluid and sodium retention and to induce peripheral vasoconstriction, which, in turn, would increase cardiac workload. Therefore, elevated plasma cortisol level may interfere with mineral corticoid activity reducing the beneficial effect of aldosterone antagonists. Thus persistent corticosteroid administration, might potentially trigger, or worsen, HF. Altered glycaemic control is also another well-known side-effect of prolonged treatment with steroids: therefore, glucose levels should be carefully monitored and, in people with diabetes, anti-diabetic treatment tailored accordingly.

At the beginning of the pandemic there were concerns that use of ACE-I or ARB medications might be associated with a greater risk of infection. However, those fears were proven to be incorrect and current evidence suggests that ACE-I or ARBs should not be stopped to prevent a COVID-19 infection or during an hospitalization in those infected, unless hypotension or worsening renal function occurs [56, 57, 58]. Careful attention should be given to hydration, fluid balance and use of diuretics: an excess of fluid administration might easily cause pulmonary oedema in those with HF, and aggressive diuresis might precipitate renal dysfunction.

A large proportion of patients admitted to hospitals receive prophylactic anticoagulants, regardless of their initial diagnosis. Observational studies suggest that anticoagulation therapy might be beneficial in patients with COVID-19, and several randomized trials are ongoing to test optimal dose and duration of thromboprophylaxis in these patients [59, 60]. However, it should be noted that haemorrhage might be as common as thrombotic events in hospitalized patients with COVID-19; therefore, although prophylactic dose anticoagulation should be prescribed, unless contraindicated, in hospitalized patients with COVID-19, and indiscriminate use of anticoagulants, especially at high doses, should be avoided.

In the setting of CVD risk or known disease, treatment with hydroxychloroquine as with many drugs, increases QT interval and predispose to arrhythmia and potentially a greater risk of a sudden death, particularly when given in combination with azithromycin, other anti-arrhythmic drugs, or in the presence of renal dysfunction and electrolyte abnormalities [61]. The widespread use of anti-inflammatory agents, administered with macrolides and antiviral drugs such as remdesivir that are likely to impair liver function, might alter drug metabolism with additional effects on the QT interval. It is good clinical practice to monitor renal and liver function and ECG, in those with severe COVID-19, regardless of a HF diagnosis.

More specifically, Remdesivir, can be used for short time period because its hepatic toxicity. In patients with Hepatic congestion and increased central vein pressure this agent may quickly impair liver function with deleterious impact on systemic and pharmacological metabolism [62]. Tocilizumab is a recombinant monoclonal antibody against IL-6 under investigation in patients with ARDS. It reduces cytokines storm but some doubts regarding increase of thromboembolic events associated with treatment may be clarified [63]. Therefore in transplant recipients, it may decrease the effect of other immunomodulatory drugs, although not direct negative effect on cardiac function have been reported [64, 65].

For those likely to deteriorate and reach the end of life, deactivation of devices, such as an implantable cardioverter defibrillator, along with withdrawal of unnecessary medications, should be considered after discussion with patients or their representatives.

## 5. Out of Hospital Management 

**Fig. 3.**
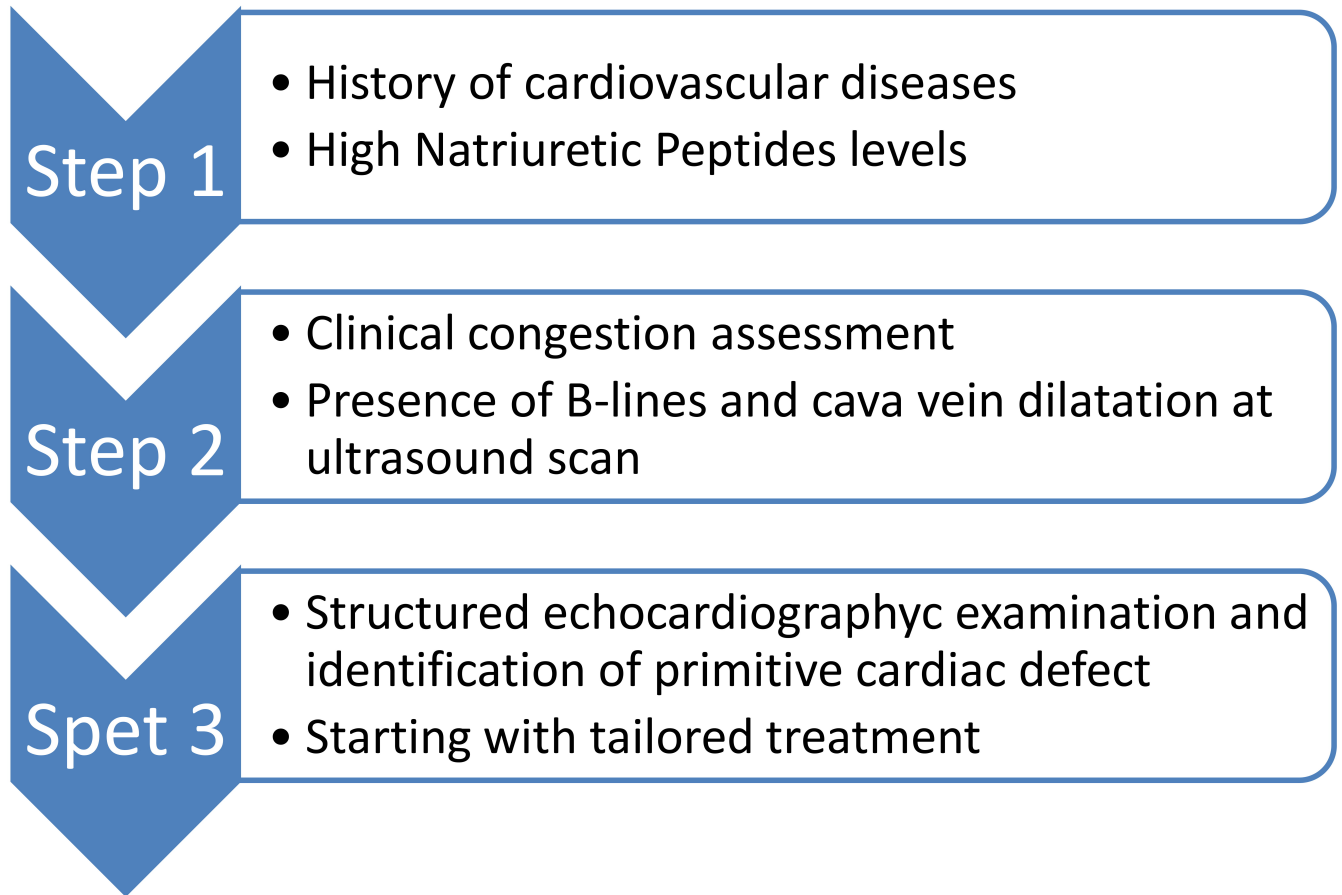
**A stepwise approach may be applied in patients with suspected HF or ARDS starting from clinical examination and blood test up to a more detailed diagnostic screening based on initial screening**.

In most part of the world, health care systems have been caught largely unprepared when invested by the COVID pandemic. On the other hand, massively supported by governments and industry, they responded rapidly, converting wards and additional external facilities to COVID-19 areas. Strict lockdown measures have been successful in controlling rate of infection, and easing pressure on hospitals. Many governments have now adopted massive testing strategies in the community to contain spread of disease, which should re-allow a gradual and safer access to diagnosis and care to those most in need [66]. Therefore the massive vaccination procedures have reduced both the needing for hospital access and the percentage of severe infection. Unfortunately, this is not enough. For months, a large proportion of patients with HF have not received any specialist input. There is a concrete possibility that many did not have their treatment optimised, have inevitably deteriorated or, eventually, died prematurely, even before an initial diagnosis was made. It is time to rapidly repristinate and reorganise HF services, to take care of a huge backlog of appointments, and recover access to diagnostic services, consultations and long-term management [67]. A wider adoption of point of care testing with natriuretic peptides in the community would identify those at greater risk of deterioration, admission to hospital and death, and therefore determine the urgency for a specialist consultation [68]. Widespread use of video consultations and home telelemonitoring would ensure a more regular and continuous delivery of care, limit travel and risk exposure to both patients and health care professionals. Recent experience in New York with a multisensor device may suggest the beneficial effects of current approach in reducing both hospitalization and infection [67]. There will be more than one creative solution for any local reality, depending on organisation and availability of resources.But facilitating access to care should be a top priority of the agenda for governments as well as for doctors, to ensure that patients with HF, with or without COVID-19, will finally receive the care they deserve [55, 69]. A specific stepwise appraoach starting from clinical history and CVD risk burden assessment,specific blood tests evaluation, then chest and cardiac ultrasound evaluation, up to a more cohomprensive echocardiographic exam including cava vein PAPS and cardiac chamber measurement, may be usefull for patients screening (Fig. 3).

## 6. Conclusions

The medical community and society as a whole has had to make dramatic adjustments in light of the COVID pandemic. There is substantial evidence that even in well-resourced, developed countries that the impact will have long-lasting effects. With the failure of the COVID-19 vaccines due to antigenic escape with the Delta and other variants, the emphasis for pandemic management has shifted to early medical treatment and concomittent therapy with AHF patients. However, there is evidence that the cardiology community will need to address the impact of COVID in relation to HF and it is clear that pre-existing cardiac conditions, in particular heart failure, leaves patients more vulnerable and they should be considered a high-risk group. Added to this, there have been substantial curtailments to elective care and changes in emergency referral patterns which has likely impacted on prognosis and will leave a substantial backlog and likely long lasting — positive and negative — shifts in the way patients are managed. The focus should now turn to gradual reinstatement of services, recovery of diagnostic and therapeutic pathways and specialist review. The transitions to remote medicine have been necessary and may be here to stay however, clinical examination and regular review should not be overlooked and a balance will need to be found moving forward.
